# Adherence to Hormonal Therapy in Breast Cancer Patients in Saudi Arabia: A Single-Center Study

**DOI:** 10.7759/cureus.24780

**Published:** 2022-05-06

**Authors:** Attiah Khobrani, Yasser Alatawi, Eshtyag Bajnaid, Omima Alemam, Abubakr Osman, Lina Bin Attash, Mohammed Jaffal, Mohammed AlGhanmi, Adnan Alharbi, Mohammed Alnuhait

**Affiliations:** 1 Pharmaceutical Care Services, King Abdullah Medical City, Makkah, SAU; 2 College of Pharmacy, University of Tabuk, Tabuk, SAU; 3 Medical Oncology, King Abdullah Medical City, Makkah, SAU; 4 College of Pharmacy, Umm Al Qura University, Makkah, SAU

**Keywords:** adjuvant tamoxifen, adjuvant hormone therapy, saudi women, early breast cancer, hormone receptor-positive breast cancer

## Abstract

Breast cancer is one of the most common types of cancer in women. Approximately three-quarters of all breast cancer patients have estrogen and/or progesterone receptor positivity. As a result, the majority of patients receive hormonal treatment for between five and 10 years. Long-term use of hormonal therapy reduces the recurrence rate and the risk of death. In Saudi patients, adherence to hormonal therapy is not adequately assessed. The primary objective of this study is to determine the clinical outcomes associated with hormonal therapy adherence in breast cancer patients.

This is a retrospective cohort study of patients who received adjuvant hormonal therapy for hormone-receptor-positive breast cancer. Patients were included if they had received at least two prescription refills following their breast cancer diagnosis. The primary outcome measure was mortality and disease progression in relation to hormonal therapy adherence. Progression of disease is defined as local recurrence or radiographic evidence of metastatic disease. The secondary outcome measure was the study population's adherence to hormonal therapy. The proportion of days covered during hormonal therapy was used to assess adherence (PDC). PDC was calculated as the number of days in the prescription period divided by the total number of days in the prescription period. Patients are considered adherent if their PDC value is greater than 0.8. The mortality and disease progression curves were generated using the Kaplan-Meier method. The proportion of patients adhering to hormonal therapy was determined using descriptive analysis. The IRB granted approval. A total of 121 patients were included in the study from the 380 patients screened. Tamoxifen, letrozole, and anastrozole were administered to 58%, 27%, and 14% of patients, respectively. The median age was 53 years. Women who were postmenopausal constituted 52.3% of the study population. The majority of patients were in Stages II and I (56.2% and 16.53%, respectively). The majority of the tumors were Grade II (58.68 %). Adherence was not associated with disease progression (HR, 0.66; 95% CI, 0.25-1.72) or mortality (HR, 1.391; 95 percent CI, 0.33-5.82). Disease progression and mortality were not found to be significantly associated with hormonal therapy adherence in this study. A larger study is required to confirm the findings of our study.

## Introduction

Breast cancer is one of the most common types of cancer, affecting approximately one-third of female cancer patients [1]. Breast cancer is one of the leading causes of death worldwide; the estimated incidence in the United States in 2019 is 268,600, accounting for 30% of all cancer cases [1]. The mortality rate of breast cancer is 12.9% [2-3]. Breast cancer accounted for 14.8% of all cancers reported in Saudi and 29.7% of all cancers reported in females in Saudi Arabia [4]. Breast cancer is being diagnosed in an increasing number of women, affecting their physical health, emotional functioning, and overall quality of life [5-6]. Numerous factors contributed to an increased risk of breast cancer, including reproductive and estrogen-related factors, obesity, endometriosis, hypertension, chest radiation exposure, dyslipidemia, family history of ovarian or breast cancer, early menarche (age 12 years), late menopause (age > 55 years), alcohol consumption, and smoking [7-8].

Breast cancer can be treated adjuvant with radiotherapy, chemotherapy, surgery, or hormonal therapy, depending on multiple factors such as genetic mutation, cancer stage, and metastatic status [9]. About three-quarters of all patients diagnosed with breast cancers are estrogen receptor-positive (ER-positive) and progesterone receptor-positive “PR-positive, consequently, most patients are offered hormonal therapy for five to 10 years [9-11]. Hormonal therapy is associated with positive outcomes leading to lower rates of disease recurrence, metastasis and decrease mortality rates, and disease-free survival [12-13]. According to National Comprehensive Cancer Network (NCCN), hormonal therapy is considered a standard of care in the treatment of women with hormone receptor-positive breast cancer [9].

Many factors play a fundamental role in the outcomes of adjuvant therapy, but perhaps one of the most critical is treatment adherence [14]. Improving medication adherence has become a priority for practitioners and manufacturers of new oral therapies in the context of improving outcomes [15]. Recently, many studies notifying poor adherence to hormonal therapy among patients with breast cancer [16-18]. Despite the positive results of hormone therapy, many women discontinue treatment prematurely for a variety of reasons, including nausea, hot flashes, insomnia, and sexual dysfunction [16-18]. However, most of the adverse reactions are not life-threatening, but they can be very distressing for some patients and affect their quality of life [11,19-20]. Adherence to hormone-based therapy is a clinically significant issue, particularly given the relatively long duration of hormone treatment. Patients must take hormonal therapy on a daily basis for five to 10 years to achieve the best possible results [9,21]. Numerous studies demonstrate statistically significant associations between non-adherence to hormonal therapy and adverse economic or medical outcomes such as cancer progression, increased inpatient days, increased total health care spending, and decreased disease-free survival [22-24].

One cross-sectional study was conducted to determine adherence to adjuvant tamoxifen in patients with breast cancer in the Saudi Arabian population. Patients who received other adjuvant hormonal therapy were excluded from this study, and 30% of the patient population who received tamoxifen had an adherence rate of less than 85%. Additionally, this study did not discuss the reasons for non-adherence to hormonal therapy or the effect of non-adherence on treatment outcomes [25]. To our knowledge, there is insufficient data on adherence to hormonal therapy and mortality among breast cancer patients in Saudi Arabia. The primary objectives of this study are to determine adherence to hormonal therapy, disease progression, and mortality in Saudi Arabian patients with breast cancer.

## Materials and methods

This is a retrospective cohort study. Data were obtained from two specialized clinics for breast cancer management. The entire study population included in this study was obtained from the hospital's breast cancer registry data, and data were obtained from King Abdullah Medical City (KAMC) using the health information system, Medica Plus. All demographic and laboratory data were available in Medica Plus and other data were obtained from patient files that were available in the medical recorded department in KAMC, for example, if they had surgery, chemotherapy, radiotherapy, or hormone therapy. Patients who visited the clinics between 2008 and 2016 were included in the study. Patients were followed in breast cancer clinics every three months. King Abdullah Medical City in Makkah is a 550-bed quaternary specialty healthcare institution in Saudi Arabia's Makkah region. The specialized hospital center is a quaternary hospital in Makkah, Saudi Arabia's Holy Capital. It is home to several centers of expertise, including cardiovascular, neurosciences, oncology, and specialized surgery. We selected research participants using convenience sampling and included all patients who satisfied the inclusion and exclusion criteria at our facility. Data from other hospitals in the area were not authorized to be accessed at the time this study was undertaken; this is one of our research's limitations. 

Inclusion criteria

The inclusion criteria were: Adult patients (18 years or more), diagnosed with breast cancer and receiving adjuvant hormonal treatment with at least two prescription refills after the breast cancer diagnosis date; non-metastatic cancer, which is Stage I, Stage II, or Stage III, hormonal receptor-positive, regardless of the number of receptors; breast cancer as the primary tumor diagnosis; and patients who had received breast cancer treatment, either surgery, chemotherapy, radiotherapy, or hormone therapy.

Exclusion criteria

The exclusion criteria were: Patients with metastatic breast cancer; patients continuing his/her treatment regimen in another health care institution; and patients starting treatment after January 1, 2017.

Adherence measurement

Hormonal therapy adherence was evaluated using the proportion of days covered (PDC). PDC was calculated as the number of days in the period covered by prescription divided by the number of days in the period. The post-index date PDC for hormonal medication was estimated for each patient. Patients were not counted twice if they filled a prescription for any drug in the same class. Adherence was defined as a PDC of ≥ 0.8 [26].

Covariates (confounding factor)

Patients’ demographic information, including age, status, and chemotherapy, were from Medica Plus, and tumor characteristics, including stage, grade, tumor size, number of presence positive lymph nodes, and estrogen receptor status, were identified in medical records and the hospital's own breast cancer registry data.

Statistical analysis

Means and proportions were used to report the patients’ baseline characteristics across cohorts, including demographic information (i.e., age, sex, marital status), tumor characteristics (i.e., stage, grade, estrogen receptor status, diagnosis year, tumor size, and some positive lymph nodes), and relevant treatments. Baseline characteristics were compared across cohorts using t-tests for continuous variables and chi-square tests for categorical variables. The models were adjusted for tumor characteristics (i.e., stage, grade, estrogen receptor status, diagnosis year, tumor size, and the number of positive lymph nodes), cancer treatments, and unbalanced covariates. The Kaplan-Meier method was used to generate the survival curves for breast cancer patients. Descriptive analysis was performed to examine the proportion of adherence to hormonal therapy. For analysis and interpretation, data were entered into IBM SPSS, version 23 (IBM Corp., Armonk, NY).

## Results

A total of 121 patients were included in the study from the 380 patients screened. Tamoxifen, letrozole, and anastrozole were administered to 58%, 27%, and 14% of patients, respectively. The distribution of baseline characteristics among women with breast cancer is shown in Table 1. Women with breast cancer who were receiving hormone therapy on average age were 53 (±13). The findings indicate that 52.34%, 42.41%, and 5.25% of women, respectively, were postmenopausal, premenopausal, or perimenopausal. The majority of patients were diagnosed with breast cancer stage II and Stage I (56.2% and 16.5%, respectively). Grade II was the highest among the study population reaching up to 58.6% followed by Grade III (17.36%) and Grade I (12.22%). Approximately 55.3%, 42.15%, and 2.48% of patients were found to have cancer on their left side, right side, and both sides, respectively. The majority of women (83.47%) tested positive for PR while 93.39% tested positive for ER. However, only 20.66% of females tested positive for human epidermal growth factor receptor 2 (HER2). Additionally, approximately 28.93% of females had lymph vascular invasion.

**Table 1:** Patients' baseline characteristics and outcomes

	All samples	Adherent	Non-adherent	P-value
Age, mean (SD)	53.9 (13.3)	53 (13.1)	56(13.8)	0.258
Menopause	N (%)			0.4026
Premenopausal	50 (42.4)	44	35.1	
Postmenopausal	62 (52.3)	47.6	59.5	
Perimenopausal	5 (5.2)	3.6	5.4	
Clinical stage	N (%)			0.1435
Stage I	20 (16.5)	21.4	5.4	
Stage II	68 (56.2)	54.8	59.5	
Stage IIIA	11 (9)	8.3	10.8	
Stage IIIB	10 (8.2)	8.3	8.1	
Grade	N (%)			0.5783
Grade I	16 (13.2)	13.1	13.5	
Grade II	71 (58.7)	59.5	56.8	
Grade III	21 (17.4)	19	13.5	
Site of cancer	N (%)			0.0753
Lift	67 (55.4)	60.7	43.2	
Right	51 (42.1)	35.7	56.8	
Bilateral	3 (2.5)	3.6	0.00	
PR status	N (%)			0.8999
Negative	17 (14)	13.1	16.2	
Positive	101 (83.5)	84.5	81	
Human epidermal growth factor receptor-2 (HER2) status	N (%)			0.8777
Negative	89 (73.5)	75	70.2	
Positive	25 (20.7)	19	24.3	
Pathology type	N (%)			0.6337
Invasive/ductal	99 (81.8)	79.76	86.49	
lobular	8 (6.6)	8.33	2.70	
Chemotherapy	N (%)			0.4178
Yes	79 (65.3)	65.5	64.9	
No	34 (28.1)	29.8	24.3	
NA	8 (6.6)	4.8	10.8	
Relapse	N (%)			0.8110
Yes	26 (21.5)	19	7	
No	95 (78.5)	65	30	
Alive	N (%)			0.6991
Yes	98 (80.9)	79	34	
No	8 (6.6)	5	3	

**Figure 1:**
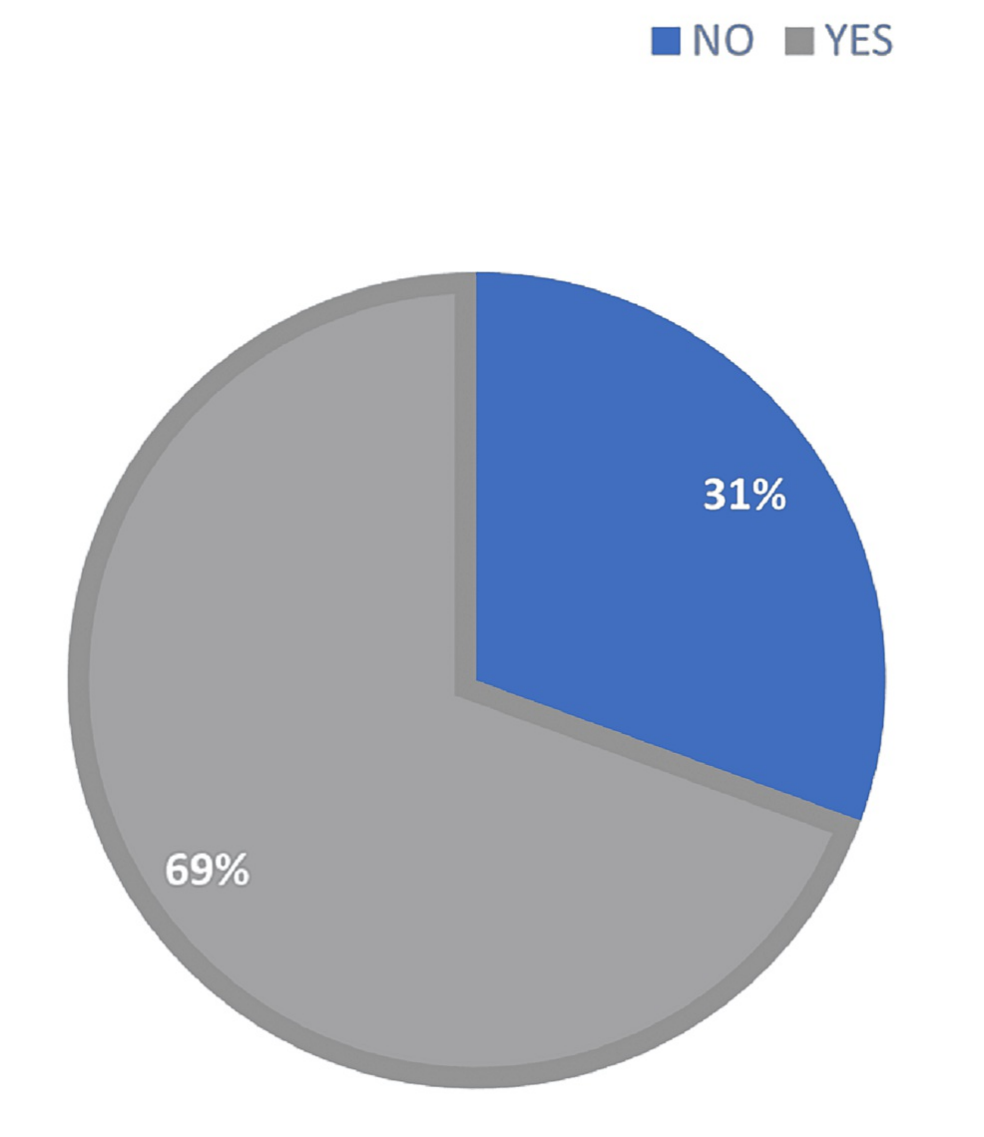
Hormonal therapy adherence

**Figure 2:**
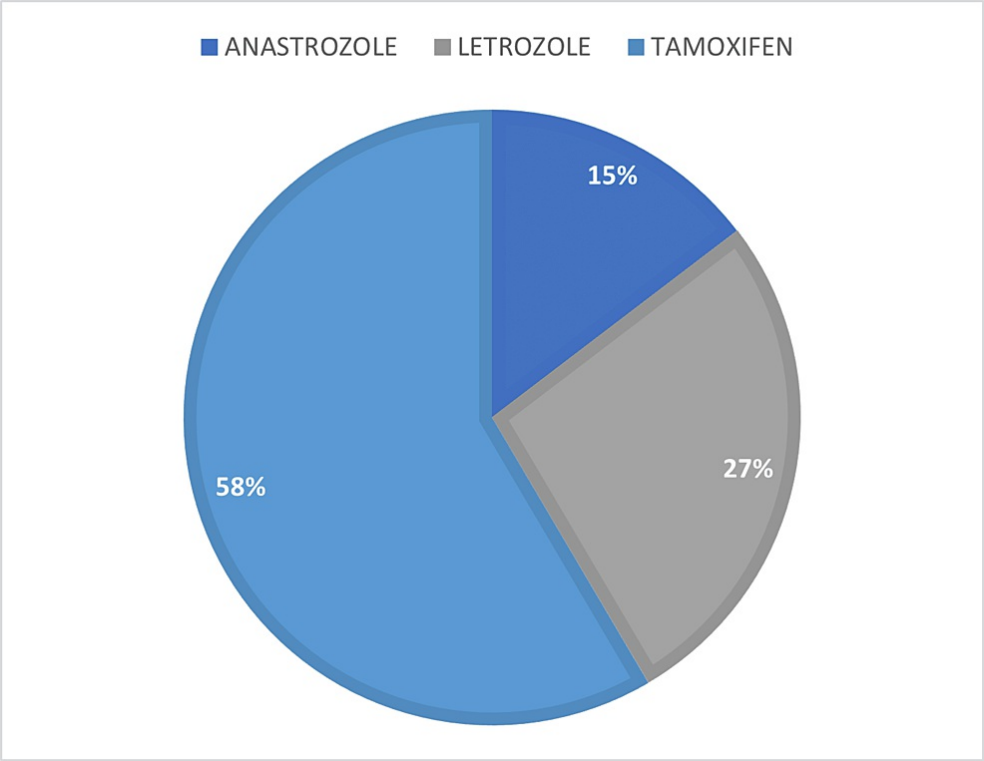
Percentage of patients taking hormone therapy

Chemotherapy was administered to the majority of women (65%). Twenty-one percent (21%) of patients experience at least one episode of relapse. Only 12.4% of patients followed throughout the study period were lost to follow-up. A total of 121 patients were enrolled during the study period; approximately 58% of patients received tamoxifen, 27% received letrozole, and 15% received anastrozole (Figures 1-2). On the other hand, 69% of patients were found to be adherent to their hormonal therapy while 31% did not meet the adherence criteria (Figures 1-2).

**Figure 3:**
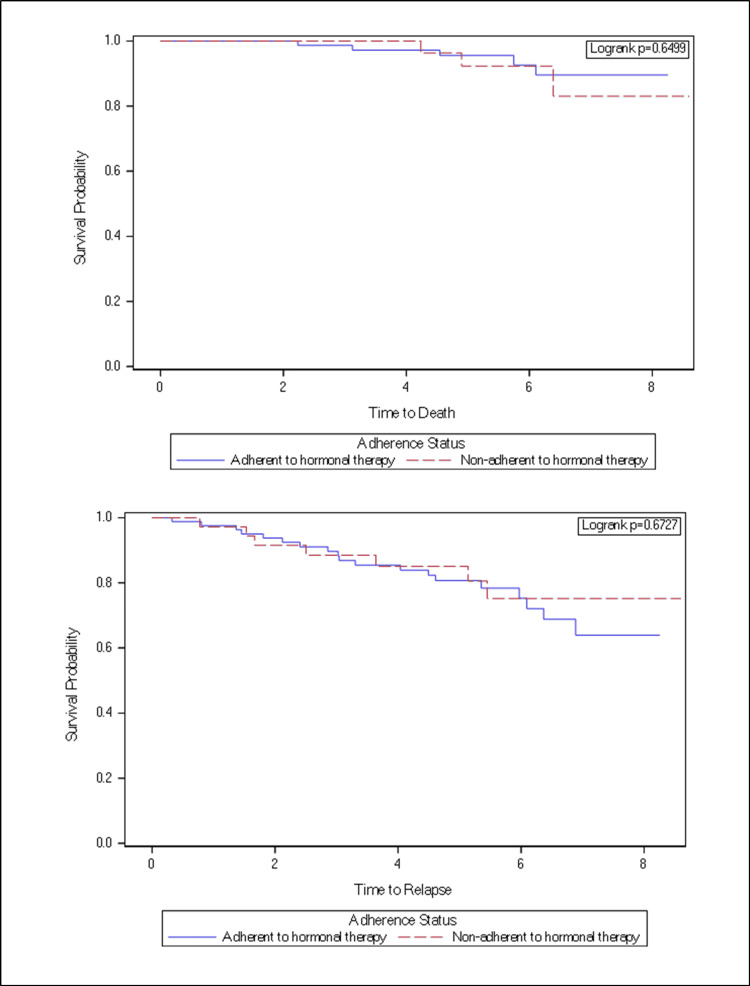
Probability of survival and relapse in women with breast cancer by adherence status

Adjusted and non-adjusted models for mortality and cancer recurrence by hormonal therapy adherence are presented in Table 2. With hormonal therapy adherence and disease progression, the adherence group shows a higher result of survival relapsed probability compared to the non-adherent group, On the other hand, the adherence group shows a higher mortality probability compared to the non-adherent group (Figure 3). Adherence did not show a significant correlation to disease progression (HR, 0.666; 95% CI, 0.257-1.725) or mortality (HR, 1.391; 95% CI, 0.332-5.828). Adherence criteria were not met in 30.58% of the study population.

**Table 2:** Hazard ratios of mortality and cancer recurrence by hormonal therapy adherence

	Hazard Ratio	95% Confidence Interval
Model 1: adjusted hazard model for mortality		
Adherence	Reference	Reference
Non-adherence	1.391	0.332 - 5.828
Model 2: adjusted hazard model for cancer recurrence		
Adherence	Reference	Reference
Non-adherence	0.666	0.257 - 1.725

## Discussion

The observed rate of adherence to hormone treatment (69%) was similar to findings from earlier published research, which suggested proportions of 60% to 72% adhere to hormonal therapy in breast cancer [27-28]. We highlight the challenges characteristic in matching certain previously published studies owing to changes in adherence definitions, research methodologies, and medication types utilized (only tamoxifen, only aromatase inhibitors, or both). We used the proportion of days covered (PDC) as the definition of measurement of adherence in our study [29]. The PDC cutoff point is the point at which treatment has a reasonable chance of having the greatest therapeutic benefit. Clinical research supports an 80% PDC threshold as a standard [29]. In our study, we used data from a single regional institution, which we recognize as a limitation. However, it is critical to underline that the hospital provides regional access, limiting admission to individuals who have already received treatment at other sites. In terms of illness stage, the discovery of decreased compliance among patients in incurable stages (III and IV) contrasts with the finding that women with bigger tumors adhered more closely [27,30]. Our study determined that there was no correlation between these characteristics. Relating to the kind of hormone treatment, additional studies have shown a low adherence rate among patients who received both tamoxifen and aromatase inhibitors [31-32]. This observation might be explained by aromatase inhibitors' frequent musculoskeletal toxicity associated with this class of medication [33]. Other studies have indicated no statistically significant differences in adherence between various forms of hormone treatment [34-35]. While anastrozole had a lower adherence rate than tamoxifen [35], it is worth noting that the majority (58%) of women in the present research cohort got tamoxifen alone while 27% received letrozole and a few women (14%) received just anastrozole.

Our observation of no association between adherence to hormone treatment and breast cancer outcomes contrasts with previous findings of strong links between adherence and breast cancer progression [36-37]. The lack of improvement in recurrence and survival with increased adherence rates in our sample could be explained by factors unique to this patient population and the breast cancers that develop in this population, the methodological limitations of refill data, and the inability to detect a potentially small effect. Our adherence metric, PDC, has been used before in studies of hormone treatment and breast cancer outcomes [38]. It is an excellent tool with minor limitations like some patients completed their prescriptions but likely did not take their medication. Second, prescription refill statistics are prone to inaccuracy due to the usage of inexpensive medicines obtained from other sources. There may be features of this patient population that account for the absence of a correlation between adherence and breast cancer recurrence or survival. Additionally, there are known lifestyle variables, such as smoking, obesity, and inactivity, which have an effect on post-breast cancer outcomes and are more prevalent in women with poor socioeconomic status [39-42]. These variables may obscure the efficacy of adjuvant hormonal therapy adherence.

Another factor to consider is patient features that impair the effectiveness of adjuvant hormonal treatment. It has been found that adverse events are a significant predictor of adherence to adjuvant hormonal therapy, such that patients experiencing fewer adverse events are more likely to adhere to treatment [43-44]. One limitation of this research is the possibility of the absence of a confounding variable, which would explain the absence of association between adherence and breast cancer outcomes, as is often the case with refill data. To minimize selection bias, we focused also on both relapse and mortality.

Finally, our ability to identify differences in breast cancer outcomes, especially cancer-free survival, may have been hindered by the sample size and/or duration of follow-up. Survival differences associated with adjuvant hormonal treatment are commonly seen at five to 10 years, but cumulative mortality reductions maybe twice as large after 15 years [13].​​​​​​​ In conclusion, we found no correlation between adjuvant hormonal therapy adherence and breast cancer recurrence or mortality in this study of breast cancer patients. A larger study is required to confirm the findings of this study. Besides that, we encourage researchers to conduct additional studies on newer oncology drugs that are taken orally and to assess drug adherence, given the limited research in this field in Saudi Arabia.

## Conclusions

According to the findings of this study, 69% of patients adhered to their hormone treatment regimen. However, it did not appear that adherence to hormonal therapy was associated with disease progression or mortality. A larger study is needed to confirm the findings of our study.

## References

[1] Siegel RL, Miller KD, Jemal A (2019). Cancer statistics, 2019. CA Cancer J Clin.

[2] Chalela P, Munoz E, Inupakutika D (2018). Improving adherence to endocrine hormonal therapy among breast cancer patients: study protocol for a randomized controlled trial. Contemp Clin Trials Commun.

[3] Antoni S, Soerjomataram I, Møller B, Bray F, Ferlay J (2016). An assessment of GLOBOCAN methods for deriving national estimates of cancer incidence. Bull World Health Organ.

[4] Alqahtani WS, Almufareh NA, Domiaty DM (2020). Epidemiology of cancer in Saudi Arabia thru 2010-2019: a systematic review with constrained meta-analysis. AIMS Public Health.

[5] Chirgwin JH, Giobbie-Hurder A, Coates AS (2016). Treatment adherence and its impact on disease-free survival in the Breast International Group 1-98 Trial of Tamoxifen and Letrozole, alone and in sequence. J Clin Oncol.

[6] Finitsis DJ, Vose BA, Mahalak JG, Salner AL (2019). Interventions to promote adherence to endocrine therapy among breast cancer survivors: a meta-analysis. Psychooncology.

[7] Chuang SC, Wu GJ, Lu YS, Lin CH, Hsiung CA (2015). Associations between medical conditions and breast cancer risk in Asians: a nationwide population-based study in Taiwan. PLoS One.

[8] Warner E (2011). Clinical practice. Breast-cancer screening. N Engl J Med.

[9] Rashmi Kumar N, Burns J, Abraham J (2021). NCCN Guidelines Version 2.2022 Breast Cancer.

[10] Roberts MC, Wheeler SB, Reeder-Hayes K (2015). Racial/Ethnic and socioeconomic disparities in endocrine therapy adherence in breast cancer: a systematic review. Am J Public Health.

[11] Davies C, Pan H, Godwin J (2013). Long-term effects of continuing adjuvant tamoxifen to 10 years versus stopping at 5 years after diagnosis of oestrogen receptor-positive breast cancer: ATLAS, a randomised trial. Lancet.

[12] Abe O, Abe R, Enomoto K (1998). Tamoxifen for early breast cancer: an overview of the randomised trials. Lancet.

[13] (2005). Effects of chemotherapy and hormonal therapy for early breast cancer on recurrence and 15-year survival: an overview of the randomised trials. Lancet.

[14] Miaskowski C, Shockney L, Chlebowski RT (2008). Adherence to oral endocrine therapy for breast cancer: a nursing perspective. Clin J Oncol Nurs.

[15] Ruddy K, Mayer E, Partridge A (2009). Patient adherence and persistence with oral anticancer treatment. CA Cancer J Clin.

[16] Neugut AI, Zhong X, Wright JD, Accordino M, Yang J, Hershman DL (2016). Nonadherence to medications for chronic conditions and nonadherence to adjuvant hormonal therapy in women with breast cancer. JAMA Oncol.

[17] Gotay C, Dunn J (2011). Adherence to long-term adjuvant hormonal therapy for breast cancer. Expert Rev Pharmacoecon Outcomes Res.

[18] Simon R, Latreille J, Matte C, Desjardins P, Bergeron E (2014). Adherence to adjuvant endocrine therapy in estrogen receptor-positive breast cancer patients with regular follow-up. Can J Surg.

[19] Van Liew JR, Christensen AJ, de Moor JS (2014). Psychosocial factors in adjuvant hormone therapy for breast cancer: an emerging context for adherence research. J Cancer Surviv.

[20] McCue DA, Lohr LK, Pick AM (2014). Improving adherence to oral cancer therapy in clinical practice. Pharmacotherapy.

[21] Goss PE, Ingle JN, Pritchard KI (2016). Extending aromatase-inhibitor adjuvant therapy to 10 years. N Engl J Med.

[22] McCowan C, Wang S, Thompson AM, Makubate B, Petrie DJ (2013). The value of high adherence to tamoxifen in women with breast cancer: a community-based cohort study. Br J Cancer.

[23] Makubate B, Donnan PT, Dewar JA, Thompson AM, McCowan C (2013). Cohort study of adherence to adjuvant endocrine therapy, breast cancer recurrence and mortality. Br J Cancer.

[24] Brito C, Portela MC, de Vasconcellos MT (2014). Adherence to hormone therapy among women with breast cancer. BMC Cancer.

[25] Arafah A, Yakout K, Rehman MU, Mohammed Alsharif A, AlJawadi MH, Al-Omar HA (2020). Prevalence of the co-prescription of tamoxifen and CYP2D6 inhibitors in Saudi population: a cross sectional study. Saudi Pharm J.

[26] Yang J, Neugut AI, Wright JD, Accordino M, Hershman DL (2016). Nonadherence to oral medications for chronic conditions in breast cancer survivors. J Oncol Pract.

[27] Kimmick G, Anderson R, Camacho F, Bhosle M, Hwang W, Balkrishnan R (2009). Adjuvant hormonal therapy use among insured, low-income women with breast cancer. J Clin Oncol.

[28] Hershman DL, Shao T, Kushi LH (2011). Early discontinuation and non-adherence to adjuvant hormonal therapy are associated with increased mortality in women with breast cancer. Breast Cancer Res Treat.

[29] (2021). PQA measures overview. https://www.pqaalliance.org/adherence-measures.

[30] Wigertz A, Ahlgren J, Holmqvist M (2012). Adherence and discontinuation of adjuvant hormonal therapy in breast cancer patients: a population-based study. Breast Cancer Res Treat.

[31] Murphy CC, Bartholomew LK, Carpentier MY, Bluethmann SM, Vernon SW (2012). Adherence to adjuvant hormonal therapy among breast cancer survivors in clinical practice: a systematic review. Breast Cancer Res Treat.

[32] Güth U, Myrick ME, Kilic N, Eppenberger-Castori S, Schmid SM (2012). Compliance and persistence of endocrine adjuvant breast cancer therapy. Breast Cancer Res Treat.

[33] Henry NL, Azzouz F, Desta Z (2012). Predictors of aromatase inhibitor discontinuation as a result of treatment-emergent symptoms in early-stage breast cancer. J Clin Oncol.

[34] Chlebowski RT, Geller ML (2006). Adherence to endocrine therapy for breast cancer. Oncology.

[35] Ziller V, Kalder M, Albert US, Holzhauer W, Ziller M, Wagner U, Hadji P (2009). Adherence to adjuvant endocrine therapy in postmenopausal women with breast cancer. Ann Oncol.

[36] Dezentjé VO, van Blijderveen NJ, Gelderblom H (2010). Effect of concomitant CYP2D6 inhibitor use and tamoxifen adherence on breast cancer recurrence in early-stage breast cancer. J Clin Oncol.

[37] McCowan C, Shearer J, Donnan PT, Dewar JA, Crilly M, Thompson AM, Fahey TP (2008). Cohort study examining tamoxifen adherence and its relationship to mortality in women with breast cancer. Br J Cancer.

[38] Sheppard VB, He J, Sutton A (2019). Adherence to adjuvant endocrine therapy in insured black and white breast cancer survivors: exploring adherence measures in patient data. J Manag Care Spec Pharm.

[39] Vona-Davis L, Rose DP (2009). The influence of socioeconomic disparities on breast cancer tumor biology and prognosis: a review. J Womens Health (Larchmt).

[40] Irwin ML, Smith AW, McTiernan A (2008). Influence of pre- and postdiagnosis physical activity on mortality in breast cancer survivors: the health, eating, activity, and lifestyle study. J Clin Oncol.

[41] Li CI, Daling JR, Porter PL, Tang MT, Malone KE (2009). Relationship between potentially modifiable lifestyle factors and risk of second primary contralateral breast cancer among women diagnosed with estrogen receptor-positive invasive breast cancer. J Clin Oncol.

[42] Holmes MD, Murin S, Chen WY, Kroenke CH, Spiegelman D, Colditz GA (2007). Smoking and survival after breast cancer diagnosis. Int J Cancer.

[43] Demissie S, Silliman RA, Lash TL (2001). Adjuvant tamoxifen: predictors of use, side effects, and discontinuation in older women. J Clin Oncol.

[44] Lash TL, Fox MP, Westrup JL, Fink AK, Silliman RA (2006). Adherence to tamoxifen over the five-year course. Breast Cancer Res Treat.

